# Editorial: Insights in musculoskeletal aging 2022

**DOI:** 10.3389/fragi.2023.1347674

**Published:** 2023-12-22

**Authors:** Kieran F. Reid

**Affiliations:** Laboratory of Exercise Physiology and Physical Performance, Brigham and Women’s Hospital, Harvard Medical School, Boston, MA, United States

**Keywords:** musculoskeletal aging, physical function, interventions, pain, osteoarthritis

This translational Research Topic focused on soliciting new insights, novel perspectives and future directions for the field of musculoskeletal aging. The Research Topic presents a unique combination of manuscripts that includes two innovative Brief Research Reports that bring focus to shoulder pain and how it can influence patient-important outcomes in older persons 1) and the role of a novel non-exercise-based approach for improving movement and posture through body awareness and whole-body postural muscle activity 2). An Original Research investigation examines, at large-scale, how nutrient deficiency can impact important bone quality parameters in older persons 3). A Review article provides a rigorous evaluation of non-modifiable factors that contribute to muscle force generation with advancing age 4). A Perspective article presents a new way of thinking about cartilage degradation and the subsequent development of osteoarthritis 5).

In their article *Shoulder Pain, Health-Related Quality of Life and Physical Function in Community-Delling Older Adults*, Davis et al. 1) reported that compared to those without shoulder pain, older adults who reported shoulder pain had inferior shoulder function and significantly reduced quality of life based on physical health and emotional health subscales of the SF-36 questionnaire. An important aspect to note about this study was that that, even though a high prevalence of the enrolled participants reported shoulder pain (>40%), this was not a sample of participant that were seeking medical treatment for their shoulder symptoms. These interesting preliminary findings should be examined in larger scales studies which may provide important insights into what appears to be a highly prevalent but underrecognized factor that may contribute to reduced physical functioning and quality of life with advancing age.

Johnson and Cohen 2) in their article *Altered Coordination Strategies during Upright Stance and Gait in Teachers of the Alexander Technique* report on an educational method that has a growing body of research indicating that it may positively impact balance and mobility, reduce join pain and reduce the physically disabling impact of neurodegenerative disorder in older adults. The Alexander Technique is a non-exercise-based approach to improve movement and posture through principles that include improving body awareness and the use of mental commands or imagery. The authors found that, compared to controls, Alexander Technique Practitioners had better, more coordinated movement patterns and postural control. Further studies to examine the potential of the Alexander Technique to impact the trajectory of age-related functional movement limitations and disability among older adults are particularly warranted.

In a retrospective observational study, Roy et al. 3) in their manuscript *25-OH Vitamin D Threshold for Optimal Bone Mineral Density in Elderly Patients with Chronic Kidney Disease* sought to identify threshold levels for optimal bone mineral density (BMD) in 1097 patients with hip fractures of whom 44% had chronic kidney disease (CKD). The authors found that using a cut-point of 25 (OH) D ≤ 27 ng/mL was superior to a cut-point of ≤30 ng/mL. This cut-point was also associated with substantially higher mortality odds and longer length of hospital stay. These data provide important information that may aid the design of interventions or refinement of treatment guidelines for a highly prevalent condition that is associated with major musculoskeletal fragility, weakness and adverse clinical outcomes.

Viecelli and Ewald 4) in their article *The Non-Modifiable Factors Age, Gender and Genetics Influence Resistance Exercise* present a thorough review of three distinct factors on resistance type exercise outcomes: age, gender and genetics. After a comprehensive description of the molecular and cellular changes that occur with aging, and the underlying mechanisms of the age-related decline in the force generating capacity of skeletal muscle, an insightful and structured discussion of the functional and physiological role of age, gender and genetics and their influence on the resistance exercise training stimulus is elegantly presented.

Van Gelder et at. 5) in their article *A New Look at Osteoarthritis: threshold potentials and an analogy to hypocalcemia* present us with an alternative but highly informative insight into the possible underlying mechanisms of osteoarthritis, one of the leading causes of physical disability in older adults. The authors effectively connect their biophysical insights with biomolecular findings to cogently posit that the existence of a threshold electrical potential is critical to osteoarthritis development and progression.

As Specialty Chief Editor for Frontier in Aging Musculoskeletal Aging I hope that this Research Topic will lead to new research endeavors that will continue to advance our understanding of the field of musculoskeletal aging. I also wish to thank all authors and reviewers for their exemplary contributions to this Research Topic.

